# Multiple Crises in Tabes Dorsalis

**Published:** 1910-10-29

**Authors:** 


### Medicine.

### MULTIPLE CRISES IN TABES DORSALIS.

The common crises in cases of locomotor ataxy are lightning pains in the legs and gastric attacks with acute abdominal pain and vomiting. It is rare to find renal, biliary, rectal, vesical, urethral, ureteral, colonic, laryngeal, œsophageal, or sweating crises. Nevertheless the great importance of not forgetting the possibility of locomotor ataxy being at the root of acute attacks of the nature of visceral or laryngeal crises cannot be too much insisted upon, as has been pointed out by many observers, amongst whom one would mention in particular Dr. Byrom Bramwell. In one patient of his, for instance, gastric, rectal, intestinal, and laryngeal crises all occurred when the tabes was not yet in an advanced stage. He was a married man, an engineer by occupation, forty-two years of age, who was admitted to hospital suffering from tabes, and also complained of difficulty in breathing and of a tight feeling in his throat. The tabetic symptoms commenced four years previous to his admission, and were characterised by lightning pains in the legs, followed by gastric crises; later some difficulty in walking developed and trouble in the bladder was noticed. In addition to the very typical and characteristic gastric attacks which occurred during the early stages of his illness he also suffered from rectal and intestinal crises and latterly from some of acute laryngeal type.

On examination of the patient when admitted to hospital he presented most of the characteristic features of tabes; the knee-jerks and Achilles-jerks were absent, but the pupils reacted to light and on convergence, there being no Argyll-Robertson pupil; there was analgesia over the chest, and on the ulnar side of the arms and legs; he experienced slight difficulty in walking, and had slight ataxia, and there was complete loss of sexual desire and power. The shooting pains in the legs which developed early in the disease continued at irregular intervals, but the gastric, rectal, and intestinal crises occurred only occasionally. He gave a history of having had gonorrhœa twenty years ago, but denied any secondary symptoms.

In connection with the laryngeal crises the patient experienced at irregular intervals difficulty in breathing and a feeling of spasmodic contraction in the throat, which caused him to be very much alarmed, and gave him a feeling as if he were being choked, and also a sensation of impending death.

The laryngeal crises of tabes are fortunately very rare, and occur in only a very small proportion of cases, whereas the gastric crises are much more common and are characterised by severe pain in the upper part of the abdomen, urgent vomiting, and sometimes the vomiting of blood, which may then be mistaken for that of gastric ulceration. One important point in the differential diagnosis is that between the attacks of tabetic gastric pain and vomiting the patient is perfectly well as far as the stomach is concerned; the other is to remember the routine examination of the knee-jerks and the pupils in all cases of apparent gastritis, ulceration, or dyspepsia.

In the patient under discussion the crises were attended with pain in the rectum, a frequent desire to defæcate, a feeling as if there was something in the rectum that must be forced away, by tenesmus, and the discharge of mucus. The intestinal crises from which the patient also suffered were attended with colicky pains in the abdomen, and apparently causeless diarrhœa alternating with obstinate constipation.

Laryngeal crises are a source of danger in that they may cause sudden swelling of the tissues of the larynx, and unless this fact is kept in mind one may be at a loss to explain some cases of sudden death; fortunately, however, sudden death due to these causes is extremely rare.

